# Promoting Digital Health Data Literacy: The Datum Project

**DOI:** 10.2196/60832

**Published:** 2025-01-03

**Authors:** Daniel Powell, Laiba Asad, Elissa Zavaglia, Manuela Ferrari

**Affiliations:** 1 Faculty of Law, McGill University, Montreal, QC, Canada; 2 The Douglas Research Centre, 6875 Boulevard LaSalle (Perry C3 E-3102), Montreal, QC, H4H 1R3, Canada, 1 5147616131 ext.3445; 3 Department of Psychiatry, McGill University, Montreal, QC, Canada

**Keywords:** health data, digital data, medical records, legislation, ethics, knowledge dissemination, learning health system, data bank

## Abstract

With the increased use of digital health innovations in Canadian health care, educating health care users, professionals, and researchers on the ethical challenges and privacy implications of these tools is essential. The Datum project, funded by the Fondation Barreau du Quebec, was created to help these actors better understand legal and ethical issues regarding the collection, use, and disclosure of digital health data for the purposes of scientific research, thereby enhancing literacy around data privacy. The project consists of a multimedia website divided into legislation and policy documents and narrative-based video content. Users can access the core legislation and policies governing the collection and use of health care data geared toward researchers and health practitioners. Users can also view the narrative-based video content explaining key concepts related to digital health data. The Datum project makes an original contribution to the field of law and ethics in health science research by using novel approaches, such as learning health systems and data banks, to improve equity in health care delivery and by generating multimedia content aimed at encouraging health care users to become better consumers and supporting the collective use of their data. The Datum project also promotes digital literacy as a digital communication tool, which has the significant potential to improve health outcomes, bridge the digital divide, and reduce health inequities.

## The Digital Health Landscape in Canada

The landscape of digital health data in Canada is multifaceted, encompassing issues of access, cultural relevance, policy implementation, and ethical considerations in using digital health tools in medicine [1]. There is a growing recognition of the potential for digital health innovations to meet the health care needs of Canadians, notwithstanding the challenges and complexities of integrating digital health data in health systems [2]. Public health research highlights the ethical challenges and privacy implications of using digital public health tools, emphasizing the need to find solutions while harnessing their potential [3]. Gasser et al [3] investigated the impact of using big data for health research and surveillance on the public demand for transparency, trust, and fairness. Such ethical challenges may be overcome by balancing risks and benefits to individuals and populations while educating consumers on this topic.

The Datum project aimed to support health care users, professionals, and researchers to better understand the legal and ethical implications of digital health data. There was a need for accessible ways of understanding the collection, storage, and uses of data and how data can be leveraged to meet the objectives of scientific research. Multimedia content and a library of resources were developed to achieve this end.

## Background: Genesis of the Datum Project

The word “datum,” the singular of data, originates from the Latin verb *dare*, meaning “to give.” As an act of giving, data may be viewed as not only the result or object of research but also an object arising from a relational context. When patients consent to participate in a research study, they agree to “give” researchers information about their health and health outcomes. The process of collecting, using, and aggregating this information turns it into data that involves the interests of those who originally shared it.

Digital health data are revolutionizing medicine, driving personalized and measurement-based care approaches, informing evidence-based practices, enabling population health management, facilitating early intervention services, supporting research and innovation, and transforming ways of delivering and monitoring health care [4,5]. Analyses of health data reveal the patient’s unique characteristics, risk and protective factors, and treatment responses while providing information on service outcomes [6]. This allows providers to develop more tailored and precise medical interventions, including targeted therapies and personalized treatment plans while improving the quality of services [7,8].

Influenced by the growing importance of digitalized data, recent legislative and regulatory changes require researchers to carefully consider the relational aspects of data and how data about people is collected, processed, and used. The European Union, through the General Data Protection Regulation, spearheaded early efforts to address these issues, establishing a global standard regarding the responsibilities of those who act as the custodians of data [9]. Recent legislation in the Canadian province of Quebec (eg, Law 25) recognizes the need for enhanced privacy and confidentiality policies, a clearly informed consent process for research participants, and governance frameworks outlining these policies [10].

This rapidly evolving issue calls for enhanced literacy around digital health data, empowering patients, caregivers, and providers to engage with data policies, ask questions, and increase their understanding of their privacy rights. Literacy about data privacy enables individuals to make informed decisions about the extent to which they share their personal information and to advocate for their privacy rights within the broader data governance framework [11].

It is important to empower health care users to support the collective use of data, which extends beyond individual interests, the economic value of data, and commercial use. The current shift toward broader considerations of social and collective value includes introducing democratic forms of governing data production [12]. Moreover, the digital economy should be relational such that individuals are interconnected within population-based data relations [12]. For example, learning health systems align data, technology, and care by continuously aggregating and analyzing ongoing health care data to improve future services, thereby creating a continuous feedback cycle for learning and quality improvement [13,14].

## Content of the Datum Website

The Datum project, a multimedia website hosted at McGill University, offers users easy access to core legislation and policy documents that offer an overview of the legislative and policy frameworks governing the collection and use of health care data in Canada and a multimedia section with narrative-based video content capturing realistic exchanges between patients and providers on the collection, use, and sharing of digital health data [15].

### Narrative-Based Video Content

**Figure 1.**
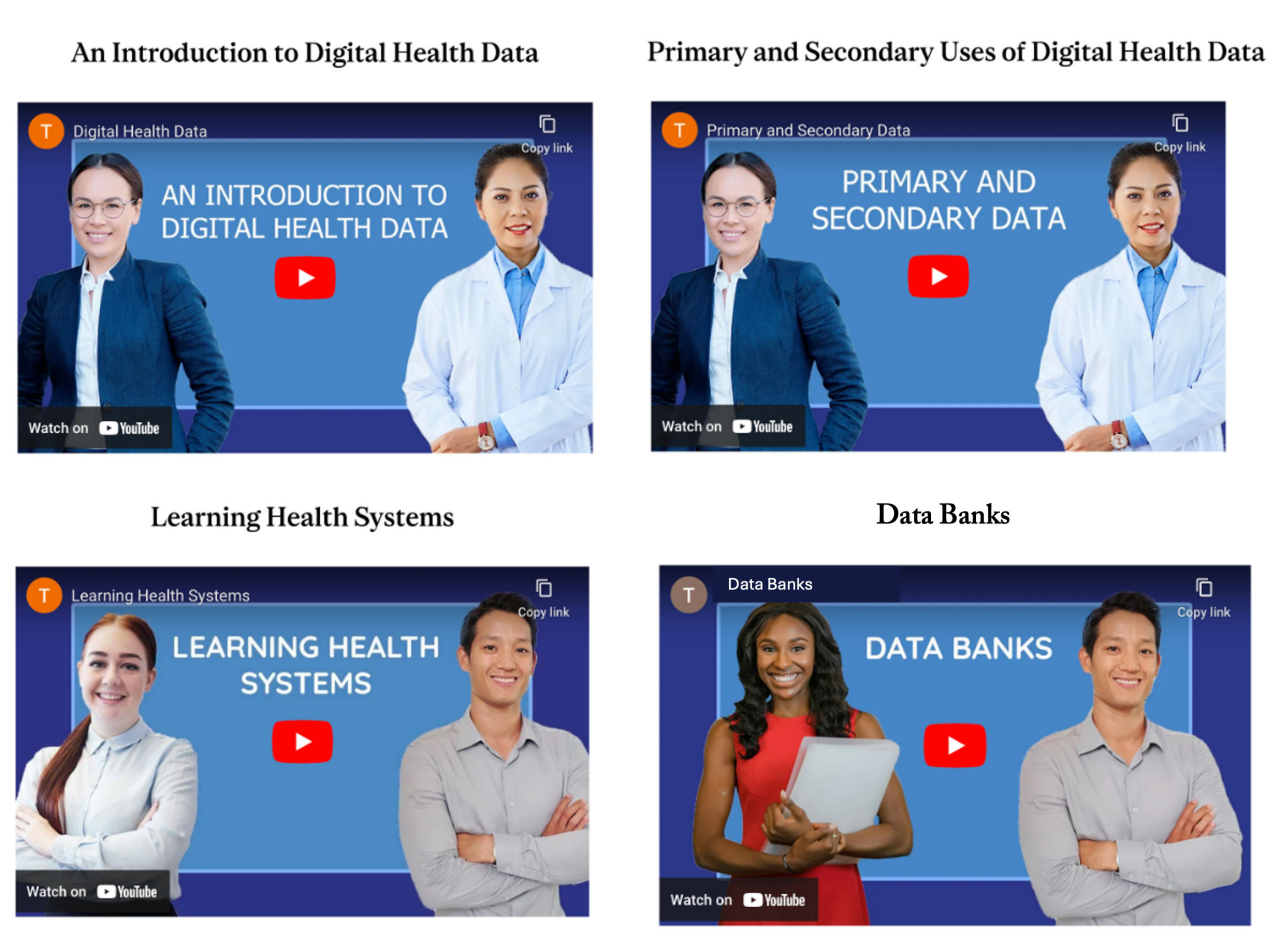
Image of 4 videos from the Datum multimedia library.

Each video animation is set in a real-world context where health care users may be asked to share their digital health data (Figure 1). For example, doctors generally gather intake information from patients seen at medical appointments, including their medical history and symptoms, then upload these data into the patients’ electronic health records. Patients who agree to participate in research studies also sign consent forms. Health care users may also identify and share concerns about sharing their personal data through dialogue with an authority figure within the health care system. Their interactions and the responses they receive demonstrate that health care providers appreciate their concerns.

By focusing on the questions of health care users regarding the collection, use, retention, and disclosure of their data in such instances, each video highlights the importance of empowering users to control their data, notably through consent and authorization, data access and transparency, revocation of consent, and data security and privacy. Through these videos, the Datum project hopes to empower health care users to make informed decisions by developing literacy in data privacy and understanding the implications of sharing digital health data in health care and research contexts. The scenarios presented also emphasize the need for collaboration and dialogue between different actors, as well as the advantages of sharing digital health data, such as informing research, empowering future research, and improving health outcomes and the delivery of health care services.

The videos build on each other to provide a comprehensive overview of the legal and ethical implications of using digital health data. The first video, “Introduction to Digital Health Data,” focuses on foundational concepts and addresses the difference between data and information. The first and second videos, which situate the characters in both clinical and research contexts, explain some foundational concepts with which health care users may be unfamiliar, such as digital health data and the difference between information and data, as well as primary and secondary uses of digital health data. The third and fourth videos present more complex systems, specifically learning health systems and data banks, where actors in the research and clinical contexts interact with each other and work together to improve health care systems and generate new knowledge. These later videos illustrate the relational aspects of data, specifically how a health care user’s data can be used for broader societal purposes. In order to develop learning health systems and data banks which rely on relational databases, health care users have a crucial need to understand the scope and potential of their consent to improve health care.

### Content Library

**Figure 2.**
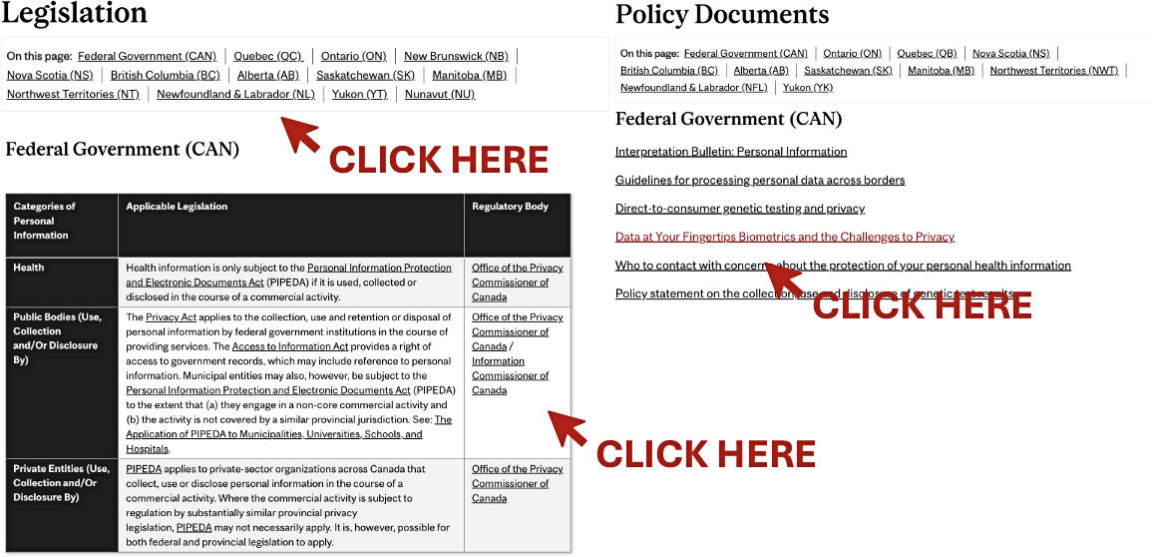
From image of how to select the legislation and policy documents Datum libraries.

As a complement to accessible multimedia content for health care users, the Datum website also features a content library, organized according to legislation and policy documents (Figure 2). This section of the website targets researchers and health care practitioners.

#### Legislation

The regulation of personal information in federalist states like Canada is governed by a complex of provincial and federal rules. The legislation page describes the division of these rules between provincial and federal authorities. At the federal level, the use, collection, and processing of personal information for commercial purposes is subject to the federal Personal Information Protection and Electronic Documents Act. In general, privacy regulation is separated according to rules for private and for public data. Health care data, which may be subject to these privacy regulations, is typically subject to additional rules. The applicable legislation for each province and territory distinguishes three categories of personal information: (1) health information (personal health care information), (2) personal information held by public bodies, and (3) personal information held by private entities.

#### Policy Documents

The policy documents page provides additional resources for user consultation regarding the collection, use, and disclosure of personal information in the health care context. These materials were selected from the publications of reputable government sources and serve as a starting point for future research.

### Feedback and Future Directions

When the Datum website was launched in September 2023, we circulated a survey to gather feedback regarding the content and accessibility of the website. User feedback suggested the utility of incorporating interactive elements into the website to increase user engagement, to allow professionals to contribute to the content library, and as ways of sharing the website with a broader audience.

### Ethical Considerations

The Datum project involves the use of fictional characters in the scenarios described in the narrative-based video content. The video content is designed to highlight foundational concepts in the literature and is based on research experience. It does not recommend specific medical procedures or decisions but rather presents interactions between these fictional characters. In addition, usability testing was used to gather feedback to improve the content of the Datum website. Survey responses were not identifiable. No personal information was collected from participants to ensure privacy and confidentiality.

## Discussion

### Contribution

The Datum project makes an original contribution to the field of law and ethics in health science research. The primary contribution of the project involved the development of literacy related to digital health data through accessible multimedia content and a focused information repository. The presentation of multimedia content transforms traditional privacy practices in the medical sphere into learning opportunities for multiple stakeholders. Digital communication tools, such as mobile health apps, telemedicine, and web-based health information resources have demonstrated a significant potential to improve health and digital literacy, ultimately leading to better health outcomes [16].

Building on the idea that digital communication tools can effectively educate and empower health care users [16], the Datum project particularly seeks to address the data-related concerns of health care users engaged in decision-making processes within the health care context. Users may be unaware of the legislative and institutional measures that protect their digital health data or the various potential uses of their data, whether at the individual or collective levels. Indeed, findings from a survey conducted by Canada Health Infoway, entitled “What Canadians Think: Exploring Public Trust in a Digital Data Ecosystem,” revealed that only 3 in 10 respondents had an awareness of Canadian privacy laws protecting their personal health information. Moreover, nearly all participants indicated that, before consenting to the use of their personal health information, they wanted to be informed of how their personal health information would be used and who would have access to it. Surprisingly, a majority of participants expressed readiness to share their personal health information for secondary purposes [17].

Further evidence supporting the promotion of digital literacy in health data, for patients to become better consumers of health care, is rapidly emerging. Informed and activated patients may effectively facilitate positive health outcomes at a lower cost [18], while digital health literacy has also been found to have a significant potential to improve health outcomes, bridge the digital divide, and reduce health inequities [19]. Studies have shown that increasing the numbers of individuals who use professional health websites is an effective measure for improving digital health literacy [20]. Additionally, research has indicated that the better their digital health literacy skills, the more individuals are able to function in the digital environment, exchange data with care providers, and actively participate in the co-design and co-delivery of health services [21]. Our rapidly evolving digital environment underlines the importance of early education in supporting the promotion of digital health literacy as an integral part of school curricula [22]. Finally, research shows that the engagement of health care service users as partners in identifying health research and service improvement priorities has led to the optimization of patient-centered health services [23].

### Limitations

Certain limitations in the Datum project should be acknowledged, especially considering the rapid rate of digital transformation. For example, the project content has yet to touch on the impact of using artificial intelligence for digital health data and challenges that the use of artificial intelligence in data modeling may create. As well, the content of the Datum project does not address the concept of data ownership, suggesting future directions for the project.

### Conclusions

The evidence from various studies supports the importance of promoting digital literacy concerning health data. The empowerment of health data users is achieved through improved health outcomes, knowledge on how to bridge the digital divide, active participation in health services, and the optimization of patient-centered care. We hope that the Datum project will empower patients to become better consumers of health care services and research.
